# Treatment outcomes and prognostic factors in patients with colorectal cancer and synchronous lung metastases in the conversion therapy era

**DOI:** 10.1007/s00384-024-04799-1

**Published:** 2025-01-09

**Authors:** Hiroaki Nozawa, Nobumi Suzuki, Tatsuya Tsushima, Koji Murono, Kazuhito Sasaki, Shigenobu Emoto, Mitsuhiro Fujishiro, Masaaki Sato, Soichiro Ishihara

**Affiliations:** 1 Department of Surgical Oncology, Graduate School of Medicine, The University of Tokyo, 7-3-1 Hongo, Bunkyo-Ku, Tokyo, 113-8655 Japan; 2 Department of Gastroenterology, Graduate School of Medicine, The University of Tokyo, Tokyo, Japan; 3 Thoracic Surgery Department, Graduate School of Medicine, The University of Tokyo, Tokyo, Japan

**Keywords:** Colorectal cancer, Lung metastases, Conversion therapy, Liver metastasis, Prognostic factor

## Abstract

**Purpose:**

The Japanese Grade Classification based on the status of pulmonary and mesenteric nodal metastases and the presence of extrapulmonary metastases had a prognostic value in patients with colorectal lung metastases previously. Because the survival of such patients has improved in the era of conversion therapy, this classification needs to be reaudited.

**Methods:**

This study reviewed the treatment sequences of 126 colorectal cancer patients with synchronous lung metastases between 2010 and 2022 at our hospital. Patients were divided into Japanese Classification Grade A, B, and C. Prognostic factors for overall survival (OS) were analyzed.

**Results:**

Thirty patients were initially diagnosed with resectable disease. Among these, 6 (35%) of 17 patients who were scheduled to undergo upfront surgery developed unresectable disease. In contrast, 3 (23%) of 13 patients receiving neoadjuvant therapy could not undergo curative resection. Twelve (13%) of 96 patients with initially unresectable metastases underwent conversion to complete resection after systemic therapy. On multivariate analysis, curative resection and H3 (> 5 liver metastases and maximum diameter > 5 cm) at diagnosis were independent prognostic factors, whereas the Japanese Grade Classification was not associated with OS.

**Conclusion:**

Instead of the Japanese classification, a new prognostic classification incorporating H3 should be established.

**Supplementary Information:**

The online version contains supplementary material available at 10.1007/s00384-024-04799-1.

## Introduction

Colorectal cancer (CRC) is commonly diagnosed and is one of the leading causes of cancer-related deaths worldwide [1]. In patients with CRC, the lung is the second most common site of metastases after the liver [2]. Even in such patients with disseminated disease, surgical resection is one of the standard treatments given the potential for prolonged overall survival (OS). In general, carefully selected patients with CRC and resectable pulmonary metastases can achieve a 5-year OS rate of approximately 30–50% [3, 4]. For patients with diseases not amenable to surgical resection, palliative systemic treatment should be considered. With the advent of efficient chemotherapeutic drugs and targeting antibodies in recent years, a new paradigm for treating stage IV CRC has emerged. For resectable liver metastasis from CRC, neoadjuvant chemotherapy (NAC) has been applied in daily clinical practice because of the EPOC trial’s report that it was associated with improved progression-free survival [5]. In unresectable colorectal liver metastasis, more intensive treatments using cytotoxic drugs along with biologics have increased the chance of conversion to curative resection and have further improved the prognosis [6]. However, no randomized controlled trials (RCT) have been conducted to test NAC in CRC with resectable lung metastasis, and only a few studies have investigated the role of conversion therapy in CRC with unresectable lung metastasis [7–9].

To predict the prognosis of patients with CRC and lung metastasis, the Japanese Society for Cancer of the Colon and Rectum uniquely developed a prognostic system in which Grades A, B, and C are determined by a matrix based on the number and laterality of lung metastases, mesenteric nodal metastasis, hepatic metastasis, and other distant-organ metastasis [10]. This prognostic classification was established based on a multicenter study in Japan on surgical cases of CRC with lung metastasis between 1991 and 2006 [11], a period before multimodal treatment strategies that enabled conversion to curative resection in patients with unresectable disease. The clinical relevance of this classification has not been systematically evaluated in patients with CRC and lung metastasis who received preoperative chemotherapy or those who underwent conversion therapy.

Therefore, the primary aim of this study was to elucidate the roles of systemic treatment in resectable or unresectable lung metastasis from CRC by reviewing the treatment sequences and outcomes in patients at our institute. The secondary aim was to investigate prognostic factors in patients with CRC and lung metastasis regardless of surgery and to judge whether the aforementioned prognostic classification still has a prognostic value in the era of conversion therapy.

## Materials and methods

### Patients

Consecutive patients with CRC and synchronous lung metastases treated between 2010 and 2022 at the University of Tokyo Hospital were retrospectively reviewed.

Primary CRC and lung metastasis were diagnosed by colonoscopy, contrast enema, and computed tomography. The resectability of primary CRC, lung and/or other organ metastases, and treatment strategy were determined by a multidisciplinary team at diagnosis and during chemotherapy periodically, discussing whether it was possible to completely resect metastases while preserving the adequate function of the target organs without sacrificing critical nearby organs [12]. Conversion was defined as the complete removal (R0 or R1) of the primary tumor and all metastatic lesions that were initially deemed unresectable.

This study was approved by the Institutional Review Board of the University of Tokyo (3252–16). The requirement for written informed consent was waived owing to the observational nature of the study.

### Data extraction

Data including sex, age, location and histology of CRC, *RAS* status, serum levels of carcinoembryonic antigen (CEA; upper limit of normal, 5 ng/mL), carbohydrate antigen (CA) 19–9 (upper limit of normal, 37 U/mL), and locoregional nodal stage at diagnosis according to the American Joint Committee on Cancer staging manual [13] were collected. In addition, the regimens and duration of systemic therapy were collected from medical charts. OS was defined as the period from the initiation of the initial treatment for colorectal lung metastasis to death.

### Classification of liver and lung metastases from CRC

Liver metastasis was classified into three categories, with H1 defined as four or fewer metastases with a maximum diameter < 5 cm, H3 as > 5 metastases including tumor(s) of 5 cm, and H2 as the remaining intermediate disease [10]. The PUL classification was determined based on the number and laterality of lung metastases, with PUL1 as one or two metastases or unilateral metastases and PUL2 as more extended lung metastasis [10]. In the case of synchronous lung metastasis, the Japanese Grade Classification is determined as follows: Grade A, PUL1, N0, and no other metastasis; Grade B, PUL1, N1/N2, and no other metastasis; and Grade C, PUL2 or the presence of extrapulmonary metastasis [10].

### Statistical analyses

All variables are summarized as medians (range), numbers (percentages), or means ± standard deviations. Categorized data were compared using the chi-square or Fisher’s exact test. Variables with a *p*-value of < 0.05 in the univariate comparison were included in a multivariate Cox regression. A multivariate Cox proportional hazards regression model was used to analyze the possible factors affecting OS. Survival curves were generated using the Kaplan–Meier method and compared using log-rank tests. Statistical analyses were performed using JMP Pro 17.0.0 (SAS Institute, Cary, NC, USA). A *p*-value < 0.05 was considered significant.

## Results

### Baseline patient profiles

During the study, 126 patients were diagnosed with CRC with synchronous lung metastasis. Table 1 summarizes the details of the patients. Among 54 patients with obstructive primary cancer, 31 underwent primary tumor resection, 17 underwent stoma creation without resection, and 2 received stent placement. The remaining four patients were allowed to have low residue diet. Eighty six patients (68%) had synchronous metastasis in organs other than the lung, with the liver being the most frequent (59%). Thirty patients (24%) had resectable CRC and lung metastasis at diagnosis. In the remaining 96 patients, the chief reason of unresectability was multiple sites of metastatic disease (36 patients), followed by peritoneal carcinomatosis (22 patients), bilateral multiple lung nodules (21 patients), bone metastasis (6 patients), inadequate function of the remaining lung after planned metastasectomy (5 patients), unresectable primary tumor (4 patients), and inability to tolerate surgery (2 patients).

**Table 1:** Clinicopathological parameters of patients

Parameter	Value
Number of patients	126
Age, years	64.9 ± 12.7
Sex, male	70 (56%)
Tumor location	
Colon	70 (56%)
Right-sided colon	32
Left-sided colon	38
Rectum	56 (44%)
Histology	
**Differentiated** adenocarcinoma	111 (88%)
**Others**	13 (10%)
N/A	2 (2%)
*RAS* status	
Wild type	71 (56%)
Mutated	40 (32%)
N/A	15 (12%)
Initial CEA level	
≤ 5 ng/mL	16 (12%)
> 5 ng/mL	109 (87%)
N/A	1 (1%)
Initial CA 19–9 level	
≤ 37 U/mL	58 (46%)
> 37 U/mL	67 (53%)
N/A	1 (1%)
Obstructive primary cancer	54 (43%)
Clinical T stage at diagnosis	
T2	2 (2%)
T3	53 (42%)
T4	71 (56%)
Clinical N stage at diagnosis	
N0	26 (21%)
N1	47 (37%)
N2	53 (52%)
Extra-pulmonary metastasis	86 (68%)
Liver metastasis*	74 (59%)
Clinical H stage at diagnosis	
H0	52 (41%)
H1	17 (13%)
H2	32 (25%)
H3	25 (20%)
Distant node metastasis*	23 (18%)
Peritoneal metastasis*	23 (18%)
Bone metastasis*	8 (6%)
Clinical PUL stage at diagnosis	
PUL1	54 (43%)
PUL2	72 (57%)
Japanese Grade Classification	
Grade A	5 (4%)
Grade B	12 (10%)
Grade C	109 (86%)
Initial resectability	
Resectable	30 (24%)
Unresectable	96 (76%)

Values are presented as the numbers of patients (%) or mean ± standard deviation

^*^Several patients had metastases in multiple organs

*N/A*, not available; *CEA*, carcinoembryonic antigen; *CA*, carbohydrate antigen

### Treatment choice and outcomes based on the initial resectability

**Fig. 1:**
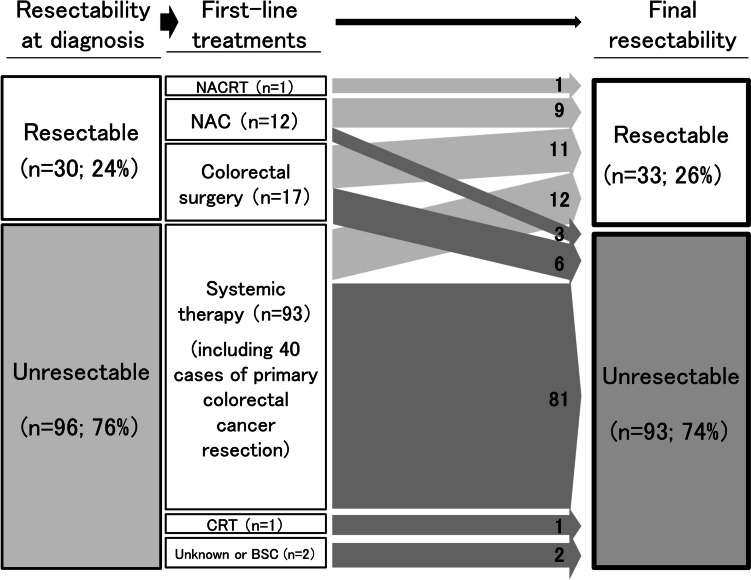
Treatment sequence and change in resectability over time. Each arrow represents each treatment sequence with the number of patients. The widths of the boxes and arrows are not proportional to the number of patients. BSC, best supportive care; CRT, chemoradiotherapy; NAC, neoadjuvant chemotherapy; NACRT, neoadjuvant chemoradiotherapy

Figure 1 illustrates the treatment sequences and the final resectability. Of the 17 patients with resectable lung metastasis from CRC who were scheduled to undergo upfront surgery with curative intent, 6 (35%) patients did not achieve complete cancer removal because they developed unresectable disease after colorectal surgery. One patient received 5-fluorouracil-based neoadjuvant chemoradiotherapy for rectal cancer following pulmonary resection of a solitary metastasis. Twelve patients received NAC before or after primary tumor resection. The details of the neoadjuvant treatments are shown in Table 2. Among patients who received neoadjuvant treatments, 3 (23%) did not achieve curative resection.

**Table 2:** Neoadjuvant treatment administered to colorectal cancer patients with resectable lung metastasis

Neoadjuvant treatment	Number of patients (%)
CAPOX	2 (6.7%)
FOLFOX	3 (10.0%)
FOLFOX + Anti-VEGF	4 (13.3%)
FOLFOX + Anti-EGFR	2 (6.7%)
SOX + Anti-VEGF	1 (3.3%)
Chemoradiotherapy	1 (3.3%)
None	17 (56.7%)
Total	30 (100.0%)

*Anti-VEGF*, antivascular endothelial growth factor; *Anti-EGFR*, antiepidermal growth factor receptor

Most patients with initially unresectable lung metastasis received systemic chemotherapy as first-line treatment without or following primary tumor resection (53 and 40 patients, respectively). One female patient received chemoradiotherapy for primary cancer of the lower rectum with bone metastases. Her condition deteriorated rapidly, and she died without receiving the intended systemic chemotherapy. After the first-line treatment, 12 (13%) patients successfully received conversion therapy (Fig. 1). The comparative details of the first-line therapy in the initially unresectable cohort stratified by conversion therapy are shown in Table 3. Most patients received an oxaliplatin-based cytotoxic drug combination as the backbone chemotherapy regimen. Targeting antibodies were included in the first-line chemotherapy regimen for all 12 patients who underwent conversion to resection. Antivascular endothelial growth factor (VEGF) antibodies were administered to approximately 60% of patients regardless of conversion, whereas antiepidermal growth factor receptor (EGFR) antibodies were more frequently (42%) used in patients who underwent successful conversion therapy than in those who did not. These differences in the first-line treatment were not statistically significant. The non-conversion group comprised more patients with right-sided colon cancer (32%) and those with wild-type RAS tumor (42%) than the conversion group (16% and 31%, respectively, Table 3). No patient with initially unresectable metastasis underwent conversion to resection after second- or later-line systemic therapy.

**Table 3:** First-line systemic therapy administered to colorectal cancer patients with unresectable lung metastasis, primary tumor location, and *RAS* status according to successful conversion to surgery

Variable	Conversion (***n*** = 12)	Non-conversion (***n*** = 84)	***p***-value
No systemic therapy/unknown	0 (0%)	2 (3%)	0.46
Chemoradiotherapy	0 (0%)	1 (1%)	
Systemic chemotherapy	12 (100%)	81 (96%)	
Chemotherapy regimen			0.81
Oxaliplatin-based	12 (100%)	63 (78%)	
Irinotecan-based	0 (0%)	3 (4%)	
Oxaliplatin + irinotecan-based	0 (0%)	8 (10%)	
Oral 5-FU regimen	0 (0%)	7 (9%)	
Biologic agents			0.11
None	0 (0%)	21 (26%)	
Anti-VEGF	7 (58%)	47 (68%)	
Anti-EGFR	5 (42%)	13 (16%)	
Tumor location			0.75
Right-sided colon	2 (16%)	27 (32%)	
Left-sided colon	5 (42%)	25 (30%)	
Rectum	5 (42%)	32 (38%)	
*RAS* status			0.72
Wild type	5 (42%)	26 (31%)	
Mutated	7 (58%)	48 (57%)	
N/A	0 (0%)	10 (12%)	

Values are presented as the numbers of patients (%)

*5-FU*, 5-fluorouracil; *Anti-VEGF*, antivascular endothelial growth factor; *Anti-EGFR*, antiepidermal growth factor receptor; *N/A*, not available

### Prognostic factors for OS

We sought prognostic factors for OS in patients with CRC and lung metastasis. We excluded a patient who did not receive any further treatment after palliative ileostomy and another patient who was transferred to another facility after palliative colostomy. Univariate analysis of the OS of the remaining 124 patients revealed that the presence of peritoneal metastasis, H3, and non-curative surgery were associated with poor OS (Table 4). Multivariate Cox proportional hazard analysis revealed that H3 was an independent prognostic factor of OS (hazard ratio [HR]: 1.86, *p* = 0.039), together with curative surgery (HR 0.15, *p* < 0.001, Table 4).

**Table 4:** Univariate and multivariate analyses of prognostic factors in colorectal cancer with lung metastasis

		Univariate		Multivariate	
Variable		HR (95% CI)	*p*-value	HR (95% CI)	*p*-value
Age, years	< 65 (vs ≥ 65)	0.67 (0.41–1.11)	0.12		
Sex	Female (vs male)	1.24 (0.75–2.04)	0.40		
Location	Right-sided colon (vs left-sided colon)	1.38 (0.70–2.77)	0.36		
	Right-sided colon (vs rectum)	1.10 (0.59–2.06)	0.77		
Histology^a^	Diff. (vs others)	0.60 (0.26–1.41)	0.24		
*RAS*^a^	Wild-type (vs mutated)	0.61 (0.35–1.07)	0.087		
CEA	Normal (vs elevated)	1.16 (0.52–2.57)	0.72		
CA 19–9	Normal (vs elevated)	0.63 (0.38–1.06)	0.080		
Obstructive primary tumor	Absent (vs present)	0.86 (0.52–1.41)	0.55		
Clinical T stage at diagnosis	T4 (vs T2/3)	1.20 (0.73–1.99)	0.47		
Clinical N stage at diagnosis	N1- (vs N0)	1.09 (0.55–2.15)	0.81		
Liver metastasis	Absent (vs present)	0.93 (0.56–1.55)	0.78		
Clinical H stage at diagnosis	H3 (vs H0-2)	2.02 (1.14–3.58)	0.016	1.86 (1.03–3.34)	0.039
Distant node metastasis	Absent (vs present)	0.88 (0.47–1.66)	0.69		
Peritoneal metastasis	Absent (vs present)	0.42 (0.24–0.75)	0.003	0.63 (0.35–1.15)	0.13
Bone metastasis	Absent (vs present)	0.50 (0.20–1.26)	0.14		
PUL stage	PUL1 (vs PUL2)	0.81 (0.49–1.34)	0.42		
Japanese Grade Classification	Grade A (vs Grade B)	2.52 (0.56–11.37)	0.23		
	Grade B (vs Grade C)	0.52 (0.19–1.44)	0.21		
Initial resectability	Resectable (vs unresectable)	0.65 (0.36–1.17)	0.15		
Curative surgery	Curative (vs non-curative)	0.14 (0.06–0.30)	< 0.001	0.15 (0.07–0.32)	< 0.001

^a^Excluding unavailable cases

*HR*, hazard ratio; *CI*, confidence interval; *CEA*, carcinoembryonic antigen; *CA*, carbohydrate antigen

### OS curve analysis

**Fig. 2:**
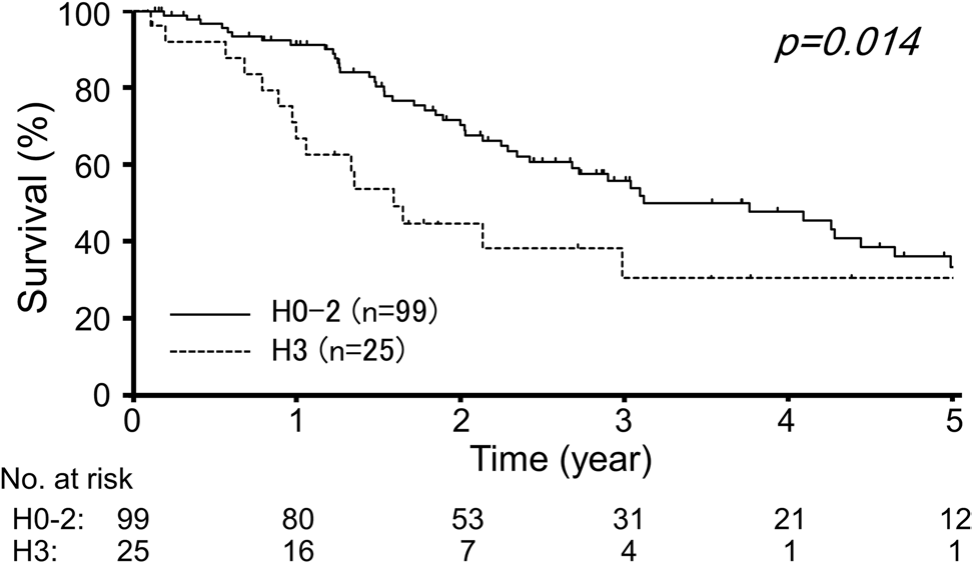
Estimated overall survival curves stratified by the initial H factor. The *p*-value was calculated by the log-rank test

**Fig. 3:**
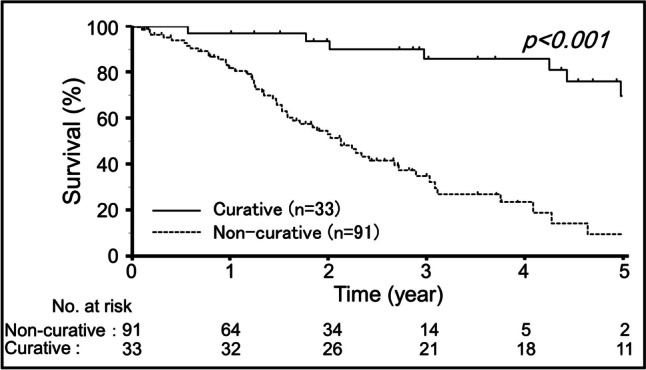
Estimated overall survival curves stratified by curative surgery. The *p*-value was calculated by the log-rank test

The OS curves of the 124 patients stratified by the independent prognostic factors identified above were analyzed. Figure 2 shows the OS curves of patients stratified by dichotomized H factor. Patients with H3 at diagnosis had a poorer survival than those with H0–H2 (3-year OS rate, 31% vs. 56%, *p* = 0.014). Figure 3 shows the OS curves of patients stratified by curative surgery. Patients who underwent curative surgery had better survival rates than those who did not (3-year OS rate, 86% vs. 35%, *p* < 0.001).

**Fig. 4:**
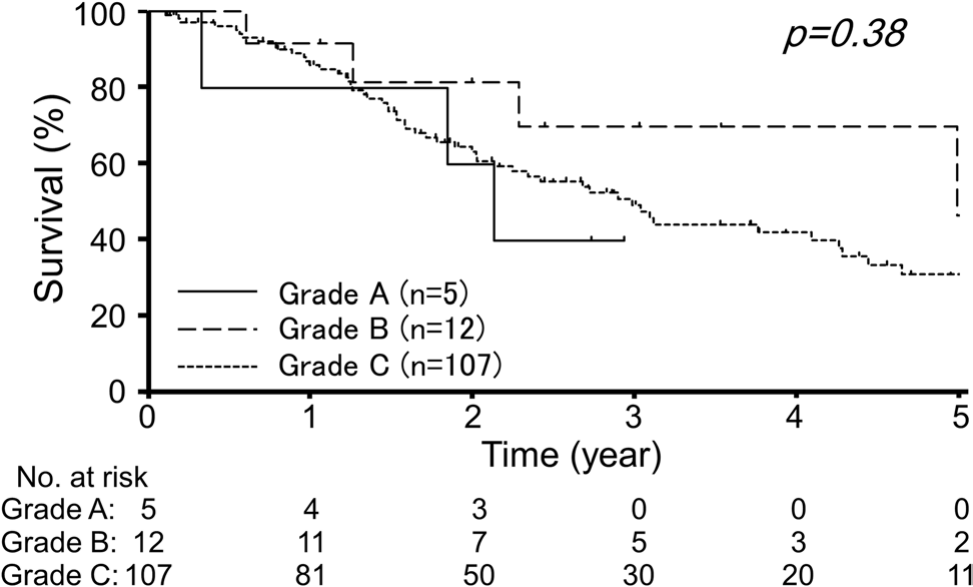
Estimated overall survival curves were compared among Grades A, B, and C for colorectal lung metastasis according to the Japanese Society for Cancer of the Colon and Rectum grade classification. The *p*-value was calculated by the log-rank test

OS was also compared according to the Japanese Grade Classification. As shown in Fig. 4, no difference in OS was observed among Grades A, B, and C. Given the small number of patients, Grades A and B (grade A + B) were combined, and OS was analyzed similarly. The OS of patients with Grade A or B did not differ from that of patients with Grade C (*p* = 0.38, Supplementary Fig. 1).

## Discussion

NAC was shown to improve DFS in resectable colorectal liver metastasis by the EPOC trial [5], whereas its roles in resectable colorectal lung metastasis remain unknown because of the absence of large-scale RCTs. In the present study, > 40% of patients with resectable CRC and synchronous lung metastasis missed the opportunity for curative resection without NAC. In contrast, the risk was reduced to < 10% when NAC was administered to patients with similar tumor burden. Therefore, NAC probably contributes to suppressing the appearance of unresectable disease in this setting.

Compared with colorectal liver metastasis, only a few studies have examined how many patients with CRC and unresectable lung metastasis can undergo conversion to surgery after systemic treatment. The probability of conversion to resection from initially unresectable disease was considered lower for lung metastasis than for liver metastasis in CRC [7, 14]. Li et al. [8] initially reported the conversion rate in unresectable lung metastasis from CRC; only 4 (5.7%) of 70 patients with CRC developed resectable disease following doublet chemotherapy regimens without targeting antibodies. We previously reported a conversion rate of 7% in patients with CRC and unresectable lung metastasis treated between 2007 and 2015 at our department [7]. In the current study analyzing patients treated during a later period at multiple departments in our hospital, the rate of successful conversion therapy was slightly raised to 12%. In the present study of patients treated during a later period at multiple departments in our hospital, the rate of successful conversion therapy was slightly increased to 12%. In a recent report from Korea, 27% of patients with CRC and unresectable lung-alone or lung and liver-limited metastasis underwent conversion surgery after FOLFIRI backbone chemotherapy plus cetuximab or bevacizumab [9]. These differences in the probability of conversion may be attributed to the types of systemic therapies and whether metastasized organs other than the lung and liver were included.

Hirosawa et al. [11] conducted a multicenter study to investigate the survival of patients with CRC and lung metastasis who underwent lung metastasectomy between 1991 and 2003, and the identified prognostic factors were further validated using different surgical cases treated between 2001 and 2006. Based on these parameters, namely, the number and laterality of lung metastases, primary nodal status, presence of liver metastasis, and extrahepatic metastasis, the Japanese Grade Classification was advocated as a prognostic system in colorectal lung metastasis in Japan [10]. However, no other prognostic classification has been published for colorectal lung metastasis. To the best of our knowledge, the present study is the first to reevaluate the prognostic performance of the Japanese Grade Classification in synchronous lung metastasis from CRC using a cohort of patients who underwent conversion therapy. As shown in Fig. 4, patients with Grade C lung metastasis showed a similar survival rate to those with Grade A or B metastasis, although the numbers of patients classified into these categories were small. Therefore, the Japanese Grade Classification may not correctly predict the prognosis of colorectal lung metastasis in the current treatment paradigm.

Researchers have investigated the prognostic factors of colorectal lung metastasis in two main settings. When only patients with CRC who underwent pulmonary metastasectomy were analyzed, the number and laterality of lung metastases, CEA level, mediastinal and hilar lymph node metastasis, aerogenous spread with floating cancer cell clusters, primary tumor stage, and past hepatic metastasis were reported as significant prognostic factors [15–18]. In contrast, previous studies on both surgical and nonsurgical cases of CRC with lung metastasis identified a distinct set of potential prognostic factors, such as conversion surgery, metachronous lung metastasis, rapid growth of metastatic deposits, and extrapulmonary metastasis [7, 19]. These variables were not composites of the Japanese Grade Classification. H3 (> 5 liver metastases including tumor(s) > 5 cm), which we newly identified as an independent prognostic factor in both resectable and unresectable CRC with synchronous lung metastasis, is not included in the matrix items for the Japanese classification. Therefore, the above variables should be considered to establish a new prognostic classification system for colorectal lung metastasis in the context of conversion therapy.

For colorectal liver metastasis, Fong et al. [20] analyzed 1001 patients who underwent metastasectomy between 1985 and 1998, reported that primary tumor N stage, disease-free interval, number and maximal diameter of metastases, and preoperative CEA level were associated with postoperative survival, and proposed the clinical risk score (CRS). Makhloufi et al. [21] analyzed 181 patients with CRC who were enrolled in the MIROX trial that compared perioperative FOLFOX4 or FOLFOX7 followed by FOLFIRI and underwent surgery for resectable liver-alone metastasis. They showed that Fong’s CRS was still a significant predictor of DFS or OS following hepatectomy [21]. On the other hand, the Japanese Grade Classification for colorectal liver metastasis was advocated [10] based on the survival analysis of 478 patients with CRC and liver metastasis who underwent hepatectomy between 1992 and 1996 [22]. The classification, comprising the size and number of liver metastases, nodule stage, and extra-hepatic metastasis, was verified to have a prognostic indicator in surgical and non-surgical cases of colorectal liver metastasis between 2007 and 2008 by a retrospective multicenter study [23]. Although two-thirds of their cohort received chemotherapy and patients with unresectable disease were included, conversion therapy had not emerged in Japan around that period. Recently, we evaluated the prognostic values of Fong’s CRS and Japanese Grade Classification for colorectal liver metastasis in a cohort of patients who underwent conversion therapy and found that both had poor discrimination abilities for OS [24]. Thus, the outdating of existing prognostic systems in the era of conversion therapy is not limited to lung metastasis from CRC but may be similarly true for metastasis in other organs including the liver.

This study had several limitations that should be acknowledged. This was a retrospective study conducted at a single hospital using a relatively small patient cohort. The treatment choice was determined at the doctors’ discretion. Only a small number of patients were categorized as having Grade A or B disease according to the Japanese classification. Recently, greater conversion rates to resectability have been achieved using intensified chemotherapy with triplet cytotoxic drugs, i.e., FOLFOXIRI, for colorectal liver metastasis [25–27]. However, only 8% of patients received the FOLFOXIRI regimen in the population with initially unresectable disease (Table 3).

This study sheds light on the importance of systemic therapy in the treatment sequence leading to complete resection of colorectal cancer with synchronous lung metastasis. Moreover, curative resection and initial H0-2 disease were significant prognostic factors in patients with colorectal lung metastasis. On the contrary, the existing Japanese grade classification had poor discrimination ability for OS. Therefore, a new classification for colorectal lung metastasis that considers the state of concomitant hepatic metastases or other prognostic parameters reported previously is warranted in the era of state-of-the-art treatments that enable conversion therapy.

## Supplementary Information

Below is the link to the electronic supplementary material.

ESM 1 Supplementary Fig. 1 Estimated overall survival curves were compared between patients with Grade A or B and those with Grade C colorectal lung metastasis according to the Japanese Society for Cancer of the Colon and Rectum grade classification. The p-value was calculated by the log-rank test (PNG 80 KB)

Supplementary file 1 (TIF 917 KB)

## Data Availability

The data that support the findings of this study are available on request from the corresponding author.
