# Supplementary figures and images for: Treatment outcomes and prognostic factors in patients with colorectal cancer and synchronous lung metastases in the conversion therapy era
Source: Int J Colorectal Dis. 2025 Jan 9;40(1):9. doi: 10.1007/s00384-024-04799-1 (PMC11711644; doi:10.1007/s00384-024-04799-1)

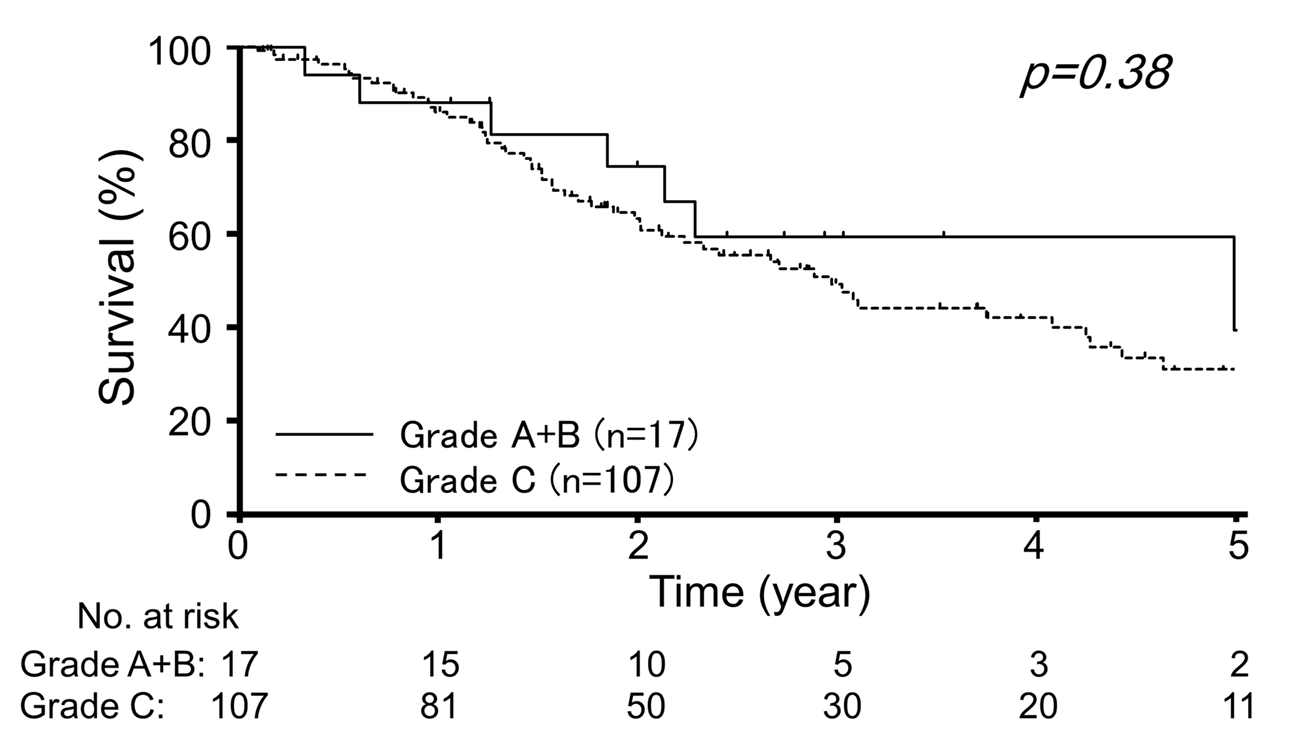

Supplement: Supplementary file 1 — Supplementary Fig. 1 Estimated overall survival curves were compared between patients with Grade A or B and those with Grade C colorectal lung metastasis according to the Japanese Society for Cancer of the Colon and Rectum grade classification. The p-value was calculated by the log-rank test (PNG 80 KB) [file 384_2024_4799_Fig5_ESM.png]

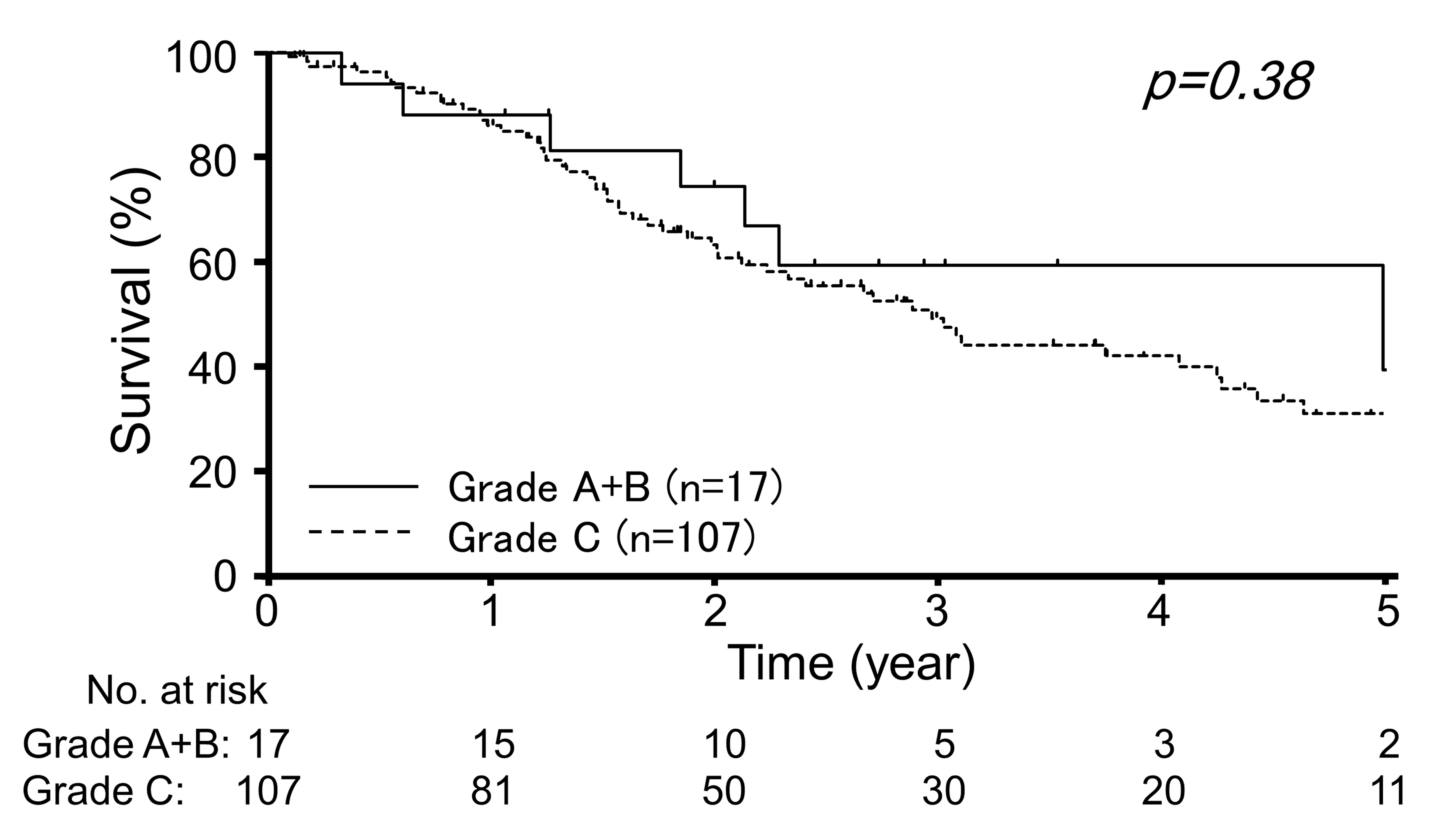

Supplement: Supplementary file 2 — Supplementary file 1 (TIF 917 KB) [file 384_2024_4799_MOESM1_ESM.tif]
